# Tropomyosin receptor kinase B-mediated signaling in integration of neuropathic pain and obesity in diabetic polyneuropathy

**DOI:** 10.31744/einstein_journal/2021AO6256

**Published:** 2021-09-17

**Authors:** Tamara Filimonova, Yulia Karakulova

**Affiliations:** 1 Perm State Medical University PermPerm Region Russia Perm State Medical University, Perm, Perm Region, Russia.

**Keywords:** Diabetic neuropathies, Brain-derived neurotrophic factor, Receptor, trkB, Obesity

## Abstract

**Objective:**

To assess the quantitative serum levels of tropomyosin receptor kinase receptor B, and to estimate its association with serum concentration of brain-derived neurotrophic factor and obesity in patients with painful and painless forms of diabetic polyneuropathy.

**Methods:**

We examined 70 patients with diabetic polyneuropathy with confirming peripheral nerve dysfunction by electroneuromyography and measuring of serum levels tropomyosin receptor kinase receptor B and brain-derived neurotrophic factor by enzyme immunoassay. Diabetic polyneuropathy was diagnosed using the modified Toronto Consensus (2011) criteria, while neuropathic pain was assessed using an 11-point Numerical Pain Rating Scale. The patients were divided into two groups according to presence or absence of neuropathic pain. Control Group consisted of 14 healthy persons.

**Results:**

The serum levels of tropomyosin receptor kinase receptor B and brain-derived neurotrophic factor in patients with diabetic polyneuropathy are significantly higher than healthy controls (p=0.000). Hyperexpression of brain-derived neurotrophic factor in serum was associated with painful form of diabetic polyneuropathy (R=0.392, p=0.012) and obesity (R=0.412, p=0.001). On the contrary high concentration of tropomyosin receptor kinase receptor B in serum associated with painless diabetic polyneuropathy by Pain DETECT (R=-0.354, p=0.015), low body weight (R=-0.354, p=0.015) and severe demyelization of nerve fibers (R=-0.574, p=0.001), indicated “non-working” receptor detected in serum.

**Conclusion:**

Tropomyosin receptor kinase receptor B signaling is involved in the modulation of neuropathic pain and obesity in diabetic polyneuropathy.

## INTRODUCTION

Neuropathic pain is a worldwide medical and social problem, associated with anxiety, depression, and low quality of life. The global prevalence of neuropathic pain varies from 6.9% and 10%, according to an ethnicity, sex and age.^(^^1^^)^ Frequency of neuropathic pain in diabetic polyneuropathy ranges from 10 to 26%.^(^^2^^)^ Meta-analyses showed the development of neuropathic pain depends on genetic mutation related to methylglyoxal-dependent modifications of voltage-gated sodium channels, in lesser degree on unmyelinated C- fiber damage, and is unrelated to demographic, anthropometric factors, duration of diabetes and a level of glycated hemoglobin.^(^^3^^–^^5^^)^ The current management of diabetic polyneuropathy (DPN) is still unsuccessful, both in preventing its development, and in stopping and modifying its progression.^(^^6^^,^^7^^)^

There are many investigations assessing the role of neurotrophic factors in development of neuropathic pain. Although brain-derived neurotrophic factor (BDNF) can play an important role in development of neuropathic pain, the pathophysiological mechanisms of its hyperalgesia effects are not clear yet.^(^^7^^,^^8^^)^ The type of receptor involved in triggering pain is still a subject ofscientific debate. Two types of receptors interacting with BDNF have been described. The first is the pan-neurotrophin receptor (p75), responsible for apoptotic mechanisms. It can bind all members of neurotrophin family and has low affinity to BDNF. The second is tropomyosin receptor kinase B (TrkB), which has high affinity for BDNF receptor. It is located on the cell membrane - the intracellular part represents tyrosine kinase domain, and the extracellular part contains ligand-binding site. After binding to specific ligand, phosphorylation of tyrosine kinase starts and activates intracellular signaling of cell survival, growth and protection of the neuron.^(^^9^^)^

But some studies revealed many functions of these signaling ways. In particular, it was verified that activation of the phosphatidylinositol-3 kinase/Akt (PI3K/Akt) signaling pathway is involved in both inflammatory processes and obesity.^(^^10^^)^ We assume all these mechanisms can be interconnected, especially in the pathogenesis of DPN, and the key point may be activation of BDNF-TrkB system.

The participation of BDNF in development of obesity and food intake regulation is a controversial issue. On the one hand, increased expression of BDNF as well as mutations in BDNF or TrkB gene are associated to obesity conditions.^(^^11^^)^ On the other hand, some research showed high positive correlations among serum levels of BDNF and body mass index (BMI), percentage of body fat, triglyceride and fasting glucose levels.^(^^12^^)^ An animal model experiment demonstrated mechanical, thermal, inflammatory nociception was reduced in the group of animals deprived of food, which correlated with reduced production of CREB, ERK and mTOR proteins in the dorsal spinal ganglion and spinal cord.^(^^13^^,^^14^^)^

The biological actions of BDNF-TrkB system in painful diabetic peripheral neuropathy in humans are still poorly understood. Serum level of TrkB in DPN patients has not been investigated yet.

## OBJECTIVE

To assess the quantitative serum levels of tropomyosin receptor kinase B, and to estimate its association with serum levels of brain-derived neurotrophic factor and obesity, in patients with painful and painless forms of diabetic polyneuropathy.

## METHODS

### Subjects

We examined 70 patients with confirmed diabetic polyneuropathy. A one-stage observational, “case-control” study was performed in 2019. The study was carried out in compliance with the ethical principles of medical research, as provided by the Declaration of Helsinki of the World Health Organization (WHO). The Ethics Committee of Perm State Medical University of the Russian Ministry of Health, approved the research, permission number 13. The Patient Group comprised 58 patients with type 2 *diabetes mellitus* (T2DM), and 12 diagnosed as type 1 *diabetes mellitus* (T1DM). There were 47 women and 23 men. The age of patients ranged from 24 to 76 years, median age of 56.75±10.88 years. The mean duration of *diabetes mellitus* was 13.05±8.41 years. These patients were on standard hypoglycemic therapy. The mean BMI of patients was 28.59±4.31kg/m^2^.

The Control Group consisted of 14 healthy individuals aged 32 to 78 years, matched for age and sex to the Patient Group. The mean age was 55.43±13.71 years, very similar to the Patient Group (U=1.381, p=0.167). The group comprised 9 women and 5 men. The mean BMI was 26.81±3.44kg/m^2^. Informed consent was obtained from each subject.

Exclusion criteria were central nervous system lesions, other types of pathology of peripheral nervous system (chronic inflammatory demyelinating polyneuropathy, carpal tunnel syndrome, asymmetric neuropathies, etc.), peripheral vascular disease, arthritis, malignancy, alcohol abuse, spinal canal stenosis, acute stage of inflammatory diseases, and pregnancy.

### Study design

Based on clinical evaluation and results of additional tests, all DPN patients were divided into two groups according to presence or absence of neuropathic pain, according to the definition of the International Association for the Study of Pain (IASP/) Neuropathic Pain Special Interest Group (NeuPSIG).^(^^1^^,^^2^^)^ The first group included 32 patients with painful DPN. A total of 38 patients with painless DPN comprised the second group.

### Severity of neuropathy

Clinical examination included assessment of patients’ complaints and analysis of medical history. Especial attention was given to symptoms that may indicate damage to the peripheral nervous system, such as burning sensation, tingling, pinching, “goosebumps”, sharp pain like “electric shock”, numbness in the feet, instability when walking, cramps in the limbs. When evaluating the medical history, we considered the factors that could trigger onset of the disease and, subsequently, interfere in progression, as well as laboratory data and instrumental examinations.

Peripheral neuropathy was evaluated according to the modified criteria of the Toronto consensus statement (2011) and the American Diabetes Association (ADA) statement on diabetic neuropathy.^(^^15^^)^ Presence and severity of peripheral polyneuropathy were assessed by the Michigan Neuropathy Screening Instrument (MNSI) and the Neurological Symptoms Score (NSS).^(^^16^^,^^17^^)^ The MNSI consists of two parts: the first part assesses subjective symptoms of peripheral polyneuropathy, including pain; the second summarizes the results of a clinical examination of the peripheral nervous system. The interpretation of MNSI is a score of 2 or more means diabetic polyneuropathy is suspected. The NSS evaluates the presence of burning, numbness, tingling, fatigue, pain, site of these signs, time since onset of symptoms, and factors that reduce severity of symptoms. The results are evaluated in points: 3-4 points correspond to mild symptoms of DPN, 5-6 points to moderate DPN, 7-9 points to severe DPN.

The neurological examination was carried out according to the Neuropathy Disability Score (NDS),^(^^18^^)^ and included a quantitative assessment of disturbances like pain, tactile, vibration perception, changes in tendon reflexes of the lower extremities, which were expressed in total scores. A Semmes-Weinstein 5.07/10 g monofilament was used for an objective assessment of tactile perception. The threshold of pain perception was assessed using a neurological hammer needle and Pin-wheel gear. Assessment of temperature perception was carried out using metal and rubberized tips of a neurological hammer. To assess the vibration perception threshold, a graduated tuning-fork with a frequency of 128Hz was employed. In normal cases, the NDS is 0-4 points. With a moderately expressed sensorimotor polyneuropathy, the score varies from 5 to 13 points. Severe polyneuropathy is diagnosed when score is over 14 points.

### Assessment of pain status

Assessment of pain status was carried out using the 11-point Numerical Pain Rating Scale (NRS),^(^^19^^)^ and a specialized screening scale, the PainDETECT questionnaire, to identify the neuropathic component of pain.^(^^20^^)^ In NRS, 0 points corresponds to no pain, and 10 points to the worst possible pain. According to PainDETECT, a total score of 0 to 12 points indicates absence of the neuropathic component of pain; from 12 to 18 points, presence of a neuropathic component of pain is possible; and a score of 18 points and more, the likelihood of a neuropathic pain is high (>90%).

### Nerve conduction studies

Peripheral nerve dysfunction was confirmed by electroneuromyography (ENMG) evaluating nerve conduction velocity (NCV), amplitude and a latency of the M-response of peroneal nerve; and amplitude, latency and NCV of the sensory S-response of sural nerve, by means of the device “Viking Quest” (Nicolet, USA). The second and third ENMG criteria, proposed by Dick et al.,^(^^21^^)^ were used as objective signs of impaired nerve conduction, as follows: a decrease of amplitude of motor response <3.5mV, NCV <40m/s, an increase in residual latency more than 3.0ms, in two or more peripheral nerves in lower extremities.

### Laboratory research

Laboratory research included assessment of degree of compensation of carbohydrate metabolism, and measuring serum levels of BDNF and TrkB by enzyme immunoassay (ELISA Kit). The degree of compensation of carbohydrate metabolism was assessed by the level of fasting blood glucose level and glycated hemoglobin (HbA1c), using the microcolumn ion exchange method, certified according to the National Glycohemoglobin Standardization Program (NGSP) or the International Federation of Clinical Chemistry (IFCC), and standardized as per the reference values adopted in the Diabetes Control and Complications Trial (DCCT).

The sandwich enzyme-linked immunosorbent assay (ELISA Kit) was performed for quantitative calculation of the brain neurotrophic factor and its high affinity for tropomyosin kinase receptor in serum, using test systems by Cloud-Clone Corp (USA). The microplate set contained antibodies conjugated with biotin, and specific for BDNF and TrkB. Samples were added to specific wells of the plate. Then horseradish peroxidase-avidin conjugate was added to each well of the plate, and incubated. After the addition of the enzyme substrate, the color changed only in the wells containing BDNF or TrkB, antibodies with biotin and horseradish peroxidase-avidin. The enzyme reaction was stopped by addition of a solution of sulfuric acid. The optical density was measured by photometric method, at a wavelength of 450±10nm. The concentration of BDNF or TrkB in samples was calculated in accordance with the standard calibration scale, prepared according to the results of analysis in a standard series. As per the instructions for ELISA, the standard curve concentrations were 10ng/mL, 5ng/mL, 2.5ng/mL, 1.25ng/mL, 0.625ng/mL,0.312ng/mL, and 0.156ng/mL.^(^^22^^)^ The minimum detectable dose of TrkB was less than 0.058ng/mL, the detection range was 0.156-10ng/mL. The minimum detectable dose of BDNF was less than 0.011ng/mL, the detection range was 0.031-10.0ng/mL. The serum levels of BDNF and TrkB obtained were expressed as ng/mL.

### Statistical analysis

Statistical analysis was performed using the Statistica version 10 software. Descriptive statistical methodswere used to calculate means and standard deviations, and nonparametric methods, in particular the Mann-Whitney U-test, to compare two independent samples from two independent groups. The threshold p<0.05 was used to assess the probability of the null hypothesis. Relations between various factors were evaluated by the Spearman correlation coefficient. The critical level of significance in testing statistical hypotheses was considered equal to 0.05. The influence of several independent variables on the dependent variable was checked by the multivariate analysis of variance (Anova - *análise multivariada de variância*). The quality of the model was evaluated by calculating R2, the square of the multiple correlation coefficient; the threshold p<0.05 was used to assess the statistical significance of the null hypothesis.

## RESULTS

Carbohydrate metabolism in patients with DPN was decompensated. The mean fasting blood glucose level was 9.01±4.09mmol/l, and it significantly exceeded the Control Group (4.78±0.57mmol/l, U=5.615, p=0.000). The mean glycated hemoglobin level was 7.62±2.11%, which is statistically higher than in the group of healthy individuals (5.04±0.36%, U=4.143, p=0.000).

The most frequent complaints presented by patients of the Patient Group included numbness, paresthesia, instability when walking, and night crumps. Pain signs were present in more than half of patients (44; 62.8%), and 32 of them had a neuropathic component (45.7%). Other study participants reporting pain symptoms had a nociceptive character of pain, mainly represented by musculoskeletal pain, low back pain and headache. The mean score of pain intensity by NRS in patients of the Patient Group was 4.58±3.21 points, which is significantly higher than in the Control Group (0.93±1.73 points, U=3.798, p=0.000). The mean severity of neuropathic pain, according to the PainDETECT scale, in the Patient Group was 13.40±10.38 points, which was also significantly higher than the mean value of the Control Group, that is, 2.50±0.85 points (U=6.348, p=0.000).

The neurological examination revealed signs of pronounced DPN in 53 patients of the Patient Group (75.7%), and severe sign of polyneuropathy was diagnosed in 17 of them (24.3%). In the Control Group, no abnormalities were observed in the peripheral nervous system upon clinical examination. The mean value of the NDS in patients with DPN was 14.98±7.12 points, which was higher in the Control Group (0.50±0.76 points, p=0.000, U=5.948).

In ENMG, all patients of the Patient Group showed signs of symmetric axonal or demyelinating polyneuropathy of varying severity, from moderate (59 cases, 84.3%) to severe (11 cases, 15.7%).

In patients with DPN, the mean serum level of BDNF determined by ELISA was significantly higher than in the Control Group (3.44±2.67ng/mL *versus* 1.07±0.64ng/mL, p=0.000). The serum level of TrkB in patients with DPN was also significantly higher than in the Control Group (4.65±2.56ng/mL *versus* 1.66±0.63ng/mL, p=0.000).

All patients with DPN were divided into two groups based on presence of neuropathic pain according to the study design. The first group included 32 patients with neuropathic pain, with mean PainDETECT score of 24.51±3.16 points, and mean NRS of 7.05±2.71 points. All patients of the first group complained of typical symptoms of neuropathic pain, localized symmetrically in the distal parts of both feet. In some patients, along with damage to nerves of the legs, the upper limbs were also involved in the pathological process. A total of 38 patients with no neuropathic pain were included in the second group; the mean PainDETECT score was 7.09±5.01, which was significantly lower, than in the first group. The mean score of MNSI in the first group significantly exceeded the corresponding parameter for patients with a painless form of neuropathy, as shown in table 1. In addition, the assessment of subjective symptoms by NSS scale in patients with neuropathic pain was significantly more pronounced than in patients with a painless form (Table 1).

**Table 1:** Clinical and neurophysiological parameters of patients with pain and non-pain

Criteria	Mean±SD Group 1	Mean±SD Group 2	U value	p value
Age, years	59.94±11.04	58.78±12.37	0.292	0.769
Body mass index, kg/m^2^	33.11±6.08*	29.49±4.88*	2.791*	0.005*
Duration of *diabetes mellitus*, years	11.00±8.64	10.40±9.48	0.418	0.675
Fasting glucose, mmol/l	8.71±2.69	8.92±4.73	0.993	0.32
HbA1c, %	7.45±2.21	7.35±2.43	0.296	0.767
NRS, points	7.05±2.71*	2.60±2.17*	3.911*	0.000*
PainDETECT, points	24.51±3.16*	7.09±5.01*	7.355*	0.000*
MNSI, points	6.85±2.06*	4.71±2.31*	3.618*	0.000*
NSS, points	6.70±2.55*	3.60±2.16*	3.045*	0.002*
NDS, points	15.88±7.71	14.75±7.49	2.445	0.064
S-amplitude sural nerve, mV	2.83±3.73	2.68±3.76	-0.087	0.930
Latency sural nerve, ms	2.82±1.79	2.87±2.49	0.185	0.852
NCV sural nerve, m/s	30.03±18.50	35.24±16.52	0.185	0.773
M-amplitude peroneal nerve, mV	1.96±1.95	1.98±2.04	0.078	0.937
Latency peroneal nerve, ms	3.55±0.96	4.02±1.44	-1.351	0.176
NCV, peroneal nerve, m/s	38.45±7.9	29.85±11.25	-0.782	0.434
BDNF, ng/mL	4.14±2.75*	2.31±1.31*	2.171*	0.029*
TrkB, ng/mL	3.11±1.76*	4.72±2.81*	3.541*	0.000*

* Significant values with p<0.05.

HbA1c: glycated hemoglobin; NRS: Numerical Rating Scale; MNSI: Michigan Neuropathy Screening Instrument; NSS: Neurological Symptom Score; NCV: nerve conduction velocity; BDNF: brain-derived neurotrophic factor; TrkB: tropomyosin receptor kinase B; SD: standard deviation.

In both groups, patients did not differ in age, duration of *diabetes mellitus*, and fasting glucose levels, and glycated hemoglobin. Patients with painful form of DPN were overweight (33.11±6.08kg/m^2^) in contrast to patients with a painless form (29.49±4.88kg/m^2^, p=0.005). Severity of polyneuropathy as per NDS did not differ in both groups. There were no differences in ENMG parameters in both groups (Table 1).

In the first group, serum levels of BDNF (4.14±2.75ng/mL) were significantly higher than in the second group (2.31±1.31ng/mL, p=0.001). However, serum levels of TrkB in first group (3.11±1.76ng/mL)were significantly lower than in second group (4.72±2.81ng/mL, p=0.005).

The correlation analysis revealed interdependences of BMI and severity of pain by NRS (R=0.315, p=0.001) and by a scale PainDETECT (R=0.327, p=0.001).

According to the statistical analysis results, age and sex of patients did not interfere in concentration of neurotrophic factor and receptor (p>0.05). Overweight was associated with high expression of serum BDNF (R=0.412, p=0.001). The inverse correlation of BMI and TrkB level was observed (R=-0.345, p=0.001).

**Figure 1:**
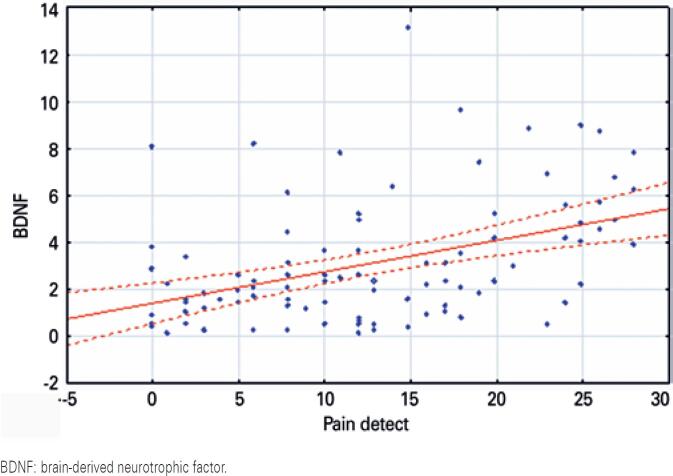
Correlation between PainDETECT and brain-derived neurotrophic factor. Pain level: serum brain-derived neurotrophic factor curve is shown in the upper right panel by scatter plot. Neuropathic pain was assessed using PainDETECT scale. The “y” axis shows serum levels of brain-derived neurotrophic factor. Serum brain-derived neurotrophic factor was measured by ELISA. The serum levels of brain-derived neurotrophic factor were expressed in ng/mL

**Figure 2:**
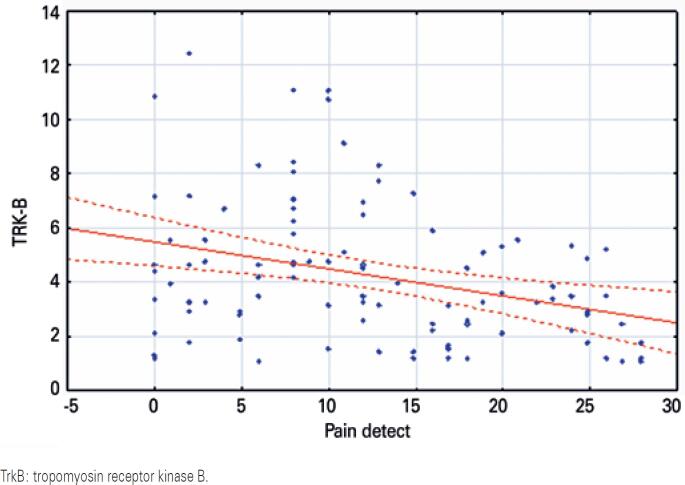
Correlation between PainDETECT and tropomyosin receptor kinase B. PainDETECT: serum tropomyosin receptor kinase B scatter plot is shown in the upper right panel and level of tropomyosin receptor kinase B is plotted on “y” axis and PainDETECT on “x” axis. Serum tropomyosin receptor kinase B was measured by ELISA. The serum levels of tropomyosin receptor kinase B were expressed in ng/mL

A correlation between severity of neuropathic pain by PainDETECT and increase in circulating BDNF (R=0.392, p=0.012), and decrease in serum TrkB (R=-0.354, p=0.015) was found, as shown in figure 1 and 2.

Fasting glycemia correlated with serum levels of BDNF (R=0.327, p=0.001). A direct correlation was found between glycated hemoglobin and serum TrkB levels (R=0.264, p= 0.001).

A direct relation between levels of BDNF and S-amplitude of the sural nerve was observed (R=0.263, p=0.016). The serum levels of TrkB were more explicitly associated with ENMG signs of impaired nerve conduction. A direct correlation between level of TrkB and latency of the peroneal nerve in distal limbs was demonstrated (R=0.311, p=0.001), as well as inverse relation in S-amplitude of the sural nerve (R=-0.235, p=0.023) and M-amplitude of peroneal nerve (R=-0.246, p=0.015, p=0.01). Besides, high serum levels of TrkB correlated with low NCV by ENMG (R=-0.574, p=0.001).

**Figure 3:**
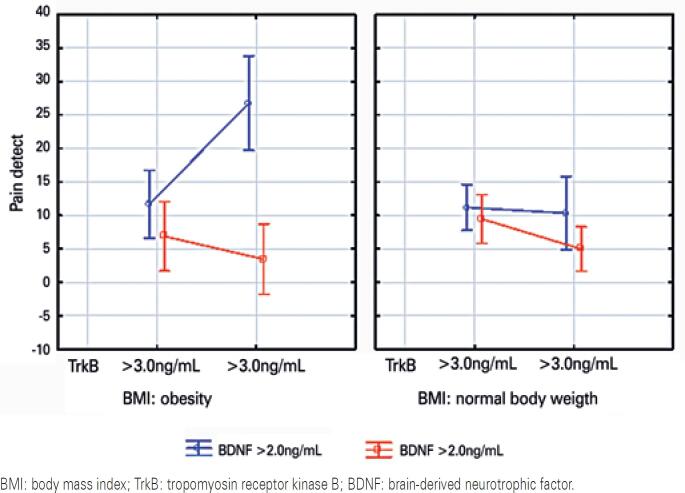
Neuropathic pain predictor model. The influence of several independent variables on the dependent variable was checked by Anova. The quality of the model was evaluated by calculating R2, the square of the multiple correlation coefficient, and the threshold p<0.05 was used to assess the statistical significance of the null hypothesis. Vertical bars denote 95% confidence intervals. The left panel shows graphs of the dependence of level of pain on the PainDETECT scale on serum levels of tropomyosin receptor kinase B and brain-derived neurotrophic factor, in obesity. The PainDETECT scale is ranked from 0 to 28 and displayed on the “y” axis. The serum levels of tropomyosin receptor kinase Bare divided into high (>3.0ng/mL) and low levels (<3.0ng/mL) and occupy the “x” axis. The blue curve shows high serum levels of brain-derived neurotrophic factor (>2.0ng/mL). The red curve corresponds to low serum levels of brain-derived neurotrophic factor (<2.0ng/mL). The right panel shows the relation of serum levels of tropomyosin receptor kinase B, depicted on the “x” axis, intensity of pain by PainDETECT on the “y”axis, and levels of brain-derived neurotrophin (red curve - brain-derived neurotrophic factor <2.0ng/mL, blue curve - brain-derived neurotrophic factor >2.0ng/mL) in cases of normal body weight. Statistical significance of the resulting models was achieved, current effect was sufficient R2=0.728, p=0.004

We analyzed the factors associated with presence and severity of neuropathic pain by Anova model. The serum levels of TrkB were divided as low (<3.0ng/mL) and high (>3.0ng/mL), and the levels of BDNF as low (<2.0ng/mL) and high (>2.0ng/mL). Obesity was diagnosed as BMI over 30kg/m^2^ according to the WHO criteria. We estimated pain intensity using PainDETECT scale. As shown in figure 3, obese patients with low serum levels of TrkB and hyperexpression of serum BDNF presented the highest intensity of neuropathic pain. The significance of the hypothesis was evaluated, the strength of the effects was sufficient (R2=0.728), and the model was statistically significant (p=0.004).

## DISCUSSION

Neuropathic pain was the primary clinical sign of DPN in 45.7% of cases according to the results of the study. Serum levels of BDNF strongly correlated with presence and intensity of neuropathic pain. Brain-derived neurotrophic factor acts as an activator of central pain sensitization, has a direct effect on modulation of neuropathic pain, and modulates its severity.

This study assessed the serum levels of TrkB in patients with diabetic polyneuropathy for the first time. Unlike its bioligand, the TrkB levels inversely relate to intensity of neuropathic pain. In addition, serum levels of TrkB strongly correlated with severity of demyelination of nerve fibers on ENMG.

We compared these correlations and the data about transmembrane localization of the receptor on myelin of peripheral nerves, as well as an impossible presence of the active free blood form of the receptor.^(^^23^^,^^24^^)^ These facts allowed us to conclude detection of TrkB in serum may indicate the circulation of a non-functioning receptor.

As previously discussed, one of the mechanisms of the central sensitizing effect of BDNF is activation of synaptic sprouting of thin sensory C-fibers in the posterior horn, which leads to hyperexcitation of glutamatergic N-methyl-D-aspartate (NMDA) receptors, development of the phenomenon of “deafferentation”, and disinhibition of neurons of somatosensory pathways.^(^^25^^–^^28^^)^ According to the results of the study, this effect is due to interaction of the brain neurotrophic factor, precisely with its functionally active high-affinity receptor TrkB. Activation of the kinase receptor triggers intracellular signals that provide neuroplasticity effects, and germination of sensory fibers.^(^^29^^,^^30^^)^ In addition, sprouting fibers with various sensitive functionalities lead to development of paresthesia, allodynia and hyperpathia.^(^^28^^,^^30^^)^ Thus, BDNF-TrkB signaling is involved in the development of neuropathic pain in DPN, and is responsible for hyperalgesia effects and transformation of nociceptive pain into neuropathic pain.

Further research is warranted to study the possibilities of controlling the cascade of intracellular signals, activated by tropomyosin receptor kinase B, in development of neuropathic pain syndrome. Scientific discoveries in this area will allow defining new vectors in pain relief in polyneuropathy.

## CONCLUSION

The study demonstrated the painful form of neuropathy is more typical for obese patients. It demonstrated overweight negatively correlated with higher levels of serum tropomyosin receptor kinase B. Hence, we assume brain-derived neurotrophic factor and its high-affinity receptor are responsible for obesity modulation. As previously discussed, inflammatory processes and obesity pathways play an important role in pathogenesis of diabetic polyneuropathy. The activation of BDNF-TrkB system can be a link in the development of hyperalgesia, obesity, and inflammation in painful form of diabetic polyneuropathy, and this connection is most likely made by phosphatidylinositol-3 kinase/Akt (PI3K/Akt) signaling pathway. A more detailed study of specific intracellular activation pathways in the development and integration of overweight, neuropathic pain and immune system is required.

Peripheral measurements of brain-derived neurotrophic factor and tropomyosin receptor kinase B provide the bases to study pathogenesis of neuropathic pain in diabetic polyneuropathy, and their action as biomarkers of the disease. Various serum levels can help differentiating pain and painless forms of diabetic polyneuropathy.
