# Supplementary material for: Tropomyosin receptor kinase B-mediated signaling in integration of neuropathic pain and obesity in diabetic polyneuropathy
Source: Einstein (Sao Paulo). 2021 Sep 17;19:eAO6256. doi: 10.31744/einstein_journal/2021AO6256 (PMC8448548; doi:10.31744/einstein_journal/2021AO6256)
Supplement: Translation

# Translation: Sinalização mediada pelo receptor de tropomiosina quinase tipo B na integração de dor neuropática e obesidade na polineuropatia diabética

**Authors:** Tamara Filimonova, Yulia Karakulova

**Affiliations:** 1 PermPerm Region Russia Perm State Medical University, Perm, Perm Region, Russia.

**Keywords:** Neuropatias diabéticas, Fator neurotrófico derivado do encéfalo, Receptor trkB, Obesidade

## Abstract

**Objetivo:**

Avaliar os níveis séricos quantitativos do receptor da tropomiosina quinase B, e estimar sua associação com os níveis séricos do fator neurotrófico derivado do cérebro e a obesidade, em pacientes com formas dolorosas e indolores de polineuropatia.

**Métodos:**

Examinamos 70 pacientes com polineuropatia diabética, com disfunção de nervo periférico confirmada por eletroneuromiografia e medida de níveis séricos do receptor da tropomiosina quinase B e do fator neurotrófico derivado do cérebro, por imunoensaio enzimático. Polineuropatia diabética foi diagnosticada através dos critérios modificados do Consenso de Toronto (2011), e a dor neuropática foi avaliada pela escala *Numerical Pain Rating* com 11 pontos. Os pacientes foram divididos em dois grupos, conforme presença ou ausência de dor neuropática. O Grupo Controle tinha 14 indivíduos saudáveis.

**Resultados:**

Os níveis séricos do receptor da tropomiosina quinase B e do fator neurotrófico derivado do cérebro em pacientes com polineuropatia diabética são significativamente mais elevados do que controles saudáveis (p=0,000). Hiperexpresssão do fator neurotrófico derivado do cérebro no soro foi associada à forma dolorosa da polineuropatia diabética (R=0,392, p=0,012) e obesidade (R=0,412, p=0,001). Por outro lado, alta concentração sérica de receptor da tropomiosina quinase B, associada à polineuropatia diabética indolor por PainDETECT (R=-0,354, p=0,015), baixo peso corporal (R=-0,354, p=0,015) e grave desmielização de fibras nervosas (R=-0,574, p=0,001), indicaram receptor “não funcionante” detectado no soro.

**Conclusão:**

A sinalização do receptor da tropomiosina quinase B está envolvida na modulação da dor neuropática e obesidade na polineuropatia diabética.

## INTRODUÇÃO

A dor neuropática é um problema médico-social global, associado à ansiedade, depressão e baixa qualidade de vida. A prevalência global da dor neuropática varia de 6,9% e 10% de acordo com a etnia, sexo e idade.^(^^1^^)^ A frequência da dor neuropática na polineuropatia diabética varia de 10 a 26%.^(^^2^^)^ As metanálises mostraram que o desenvolvimento da dor neuropática depende de mutações genéticas relacionadas a modificações dependentes de metilglioxal dos canais de sódio dependentes de voltagem, em menor grau a danos das fibras C não mielinizadas, e não está relacionado a fatores demográficos, antropométricos, duração do diabetes e nível de hemoglobina glicada.^(^^3^^–^^5^^)^ A conduta atual na polineuropatia diabética (PND) ainda não é bem sucedida, tanto na prevenção de seu desenvolvimento, como na interrupção e modificação de sua progressão.^(^^6^^,^^7^^)^

Há muitas pesquisas dedicadas ao estudo do papel dos fatores neurotróficos no desenvolvimento da dor neuropática. Apesar do fator neurotrófico derivado do cérebro (BDNF - *brain-derived neurotrophic factor*) poder desempenhar um papel importante no desenvolvimento da dor neuropática, os mecanismos fisiopatológicos de seus efeitos hiperalgésicos ainda não estão claros.^(^^7^^,^^8^^)^ O tipo de receptor envolvido no desencadeamento da dor ainda é tema de debate científico. Estão descritos dois tipos de receptores que interagem com BDNF. O primeiro é o receptor pan-neurotrofina (p75), responsável pelos mecanismos de apoptose. Pode ligar todos os membros da família das neurotrofinas e tem baixa afinidade com o BDNF. O receptor é o receptor de tropomiosina quinase B (TrkB), com alta afinidade ppelo receptor de BDNF. Está localizado na membrana celular, a parte intracelular representa o domínio da tirosina quinase, e a parte extracelular contém o sítio de ligação de ligantes. Após a ligação ao ligante específico, inicia a fosforilação de tirosina quinase, e ativa a sinalização intracelular de sobrevida celular, crescimento e proteção do neurônio.^(^^9^^)^

Mas estudos em série revelaram muitas funções destas formas de sinalização. Em particular, verificou-se que a ativação da via de sinalização do fosfatidilinositol-3 quinase/Akt (PI3K/Akt) está envolvida tanto nos processos de inflamação quanto de obesidade.^(^^10^^)^ Supomos que todos esses mecanismos possam ser interli­gados, especialmente na patogênese da PND, e o ponto-chave pode ser a ativação do sistema BDNF-TrkB.

A participação da BDNF no desenvolvimento da regulamentação da obesidade e da ingestão de alimentos é uma questão controversa. Por um lado, o aumento da expressão de BDNF bem como mutações no gene BDNF ou TrkB estão associadas às condições de obesidade.^(^^11^^)^ Por outro lado, algumas pesquisas mostraram correlações altamente positivas entre os níveis séricos de BDNF e índice de massa corporal (IMC), percentagem de gordura corporal, triglicérides e níveis de glicose de jejum.^(^^12^^)^ Um experimento com modelo animal demonstrou que a nocicepção mecânica, térmica e inflamatória foi reduzida no grupo de animais privados de alimento, o que se correlacionou com a produção reduzida de proteínas CREB, ERK e mTOR no gânglio espinhal dorsal e na medula espinhal.^(^^13^^,^^14^^)^

As ações biológicas do sistema BDNF-TrkB na neuropatia periférica diabética dolorosa em humanos ainda são mal compreendidas. O nível sérico de TrkB em pacientes PND ainda não foi investigado.

## OBJETIVO

Avaliar os níveis séricos quantitativos do receptor da tropomiosina quinase B, e estimar sua associação com os níveis séricos do fator neurotrófico derivado do cérebro e a obesidade, em pacientes com formas dolorosas e indolores de polineuropatia diabética.

## MÉTODOS

### Sujeitos de pesquisa

Examinamos 70 pacientes com polineuropatia diabética confirmada. Em 2019, foi realizado um estudo observacional de uma etapa, “caso-controle”. O estudo foi realizado em conformidade com os princípios éticos da pesquisa médica, estabelecidos pela Declaração de Helsinque da Organização Mundial da Saúde (OMS). O Comitê de Ética da *Perm State Medical University*, do Ministério da Saúde da Rússia, aprovou a pesquisa, com permissão número 13. O Grupo Pacientes tinha 58 pacientes com *diabetes mellitus* tipo 2 (T2DM), e 12 diagnosticados como *diabetes mellitus* tipo 1 (T1DM). Havia 47 mulheres e 23 homens. A idade dos pacientes variou de 24 a 76 anos, mediana de 56,75±10,88 anos. A duração média do *diabetes mellitus* foi 13,05±8,41 anos. Estes pacientes estavam em terapia hipoglicêmica padrão. O IMC médio dos pacientes foi 28,59±4,31kg/m^2^.

O Grupo Controle tinha 14 indivíduos saudáveis entre 32 e 78 anos, equiparados para idade e sexo ao Grupo Pacientes. A média de idade foi 55,43±13,71 anos, muito semelhante ao Grupo Pacientes (U=1,381, p=0,167). O grupo era composto por 9 mulheres e 5 homens. O IMC médio foi 26,81±3,44kg/m^2^. O consentimento informado foi obtido de cada participante.

Os critérios de exclusão foram lesões do sistema nervoso central, outros tipos de patologia do sistema nervoso periférico (polineuropatia inflamatória crônica desmielinizante, síndrome do túnel do carpo, neuropatias assimétricas, etc.), doença vascular periférica, artrite, doença maligna, abuso de álcool, estenose do canal espinhal, estágio agudo de doenças inflamatórias, e gravidez.

### Desenho do estudo

Com base na avaliação clínica e nos resultados de exames adicionais, todos os pacientes com PND foram divididos em dois grupos, com base em presença ou ausência de dor neuropática, de acordo com a definição de *International Association for the Study of Pain* (IASP)/*Neuropathic Pain Special Interest Group* (NeuPSIG).^(^^1^^,^^2^^)^ O primeiro grupo incluiu 32 pacientes com PND dolorosa. Um total de 38 pacientes com PND sem dor formou o segundo grupo.

### Gravidade da neuropatia

O exame clínico incluiu a avaliação das queixas dos pacientes e a análise da anamnese. Foi dada especial atenção aos sintomas que podem indicar danos ao sistema nervoso periférico, como sensação de queimação, formigamento, beliscões, “arrepios”, dor aguda como “choque elétrico”, dormência nos pés, instabilidade ao andar, cãibras nos membros. Ao avaliar a anamnese, consideramos os fatores que poderiam desencadear o início da doença e, posteriormente, interferir na evolução, bem como os dados laboratoriais e exames instrumentais.

A neuropatia periférica foi avaliada de acordo com os critérios modificados da declaração de consenso de Toronto (2011) e da declaração da *American Diabetes Association* (ADA) sobre neuropatia diabética.^(^^15^^)^ A presença e a gravidade da polineuropatia periférica foram avaliadas pelo *Michigan Neuropathy Screening Instrument* (MNSI) e pelo *Neurological Symptoms Score* (NSS).^(^^16^^,^^17^^)^ O MNSI tem duas partes: a primeira avalia os sintomas subjetivos da polineuropatia periférica, incluindo dor; a segunda resume os resultados de um exame clínico do sistema nervoso periférico. A interpretação do MNSI é uma pontuação de 2 ou mais significa que há suspeita de polineuropatia diabética. O NSS avalia a presença de queimação, dormência, formigamento, fadiga, dor, local desses sinais, tempo desde início dos sintomas, e fatores que reduzem a gravidade dos sintomas. Os resultados são avaliados em pontos: 3-4 pontos correspondem a sintomas leves de PND, 5-6 pontos a PND moderada, 7-9 pontos a PND grave.

O exame neurológico foi realizado de acordo com o *Neuropathy Disability Score* (NDS),^(^^18^^)^ e incluiu uma avaliação quantitativa de distúrbios como dor, tato, percepção de vibração, e alterações nos reflexos tendinosos das extremidades inferiores, que foram expressos em pontuação total. Um monofilamento Semmes-Weinstein 5,07/10g foi usado para uma avaliação objetiva da percepção tátil. O limiar de percepção da dor foi avaliado utilizando uma agulha de martelo neurológico e *pin-wheel gear*. A avaliação da percepção da temperatura foi realizada utilizando pontas de metal e borracha de um martelo neurológico. Para avaliar o limiar de percepção da vibração, foi empregado um diapasão graduado, com frequência de 128Hz. Em casos normais, o NDS é de 0-4 pontos. Com uma polineuropatia sensório motora moderada expressa, a pontuação varia de 5 a 13 pontos. Já a grave é diagnosticada quando a pontuação é superior a 14 pontos.

### Avaliação do estado da dor

A avaliação do estado da dor foi realizada utilizando a escala *Numerical Pain Rating* (NRS) de 11 pontos,^(^^19^^)^ e uma escala de triagem especializada, o questionário Pain- DETECT, para identificar o componente neuropático da dor.^(^^20^^)^ Na NRS, 0 pontos correspondem a nenhuma dor, e 10 pontos à pior dor possível. Segundo o PainDETECT, uma pontuação total de 0 a 12 pontos indica ausência do componente neuropático da dor; de 12 a 18 pontos, a presença de um possível componente neuropático da dor; e uma pontuação de 18 pontos ou mais, uma alta probabilidade de uma dor neuropática (>90%).

### Estudos de condução nervosa

A disfunção do nervo periférico foi confirmada pela eletroneuromiografia (ENMG) avaliando a velocidade de condução do nervo (NCV), amplitude e latência da resposta M do nervo fibular; e amplitude, latência e NCV da resposta S sensorial do nervo sural, por meio do dispositivo *“Viking Quest”* (Nicolet, EUA). O segundo e terceiro critérios para ENMG, propostos por Dick et al.,^(^^21^^)^ foram usados como sinais objetivos de com­prometimento da condução nervosa: uma diminuição da amplitude da resposta motora <3,5mV, NCV <40m/s, e um aumento da latência residual mais de 3,0ms, em dois ou mais nervos periféricos em extremidades inferiores.

### Pesquisa de laboratório

As pesquisas de laboratório incluíram a avaliação do grau de compensação do metabolismo dos carboidratos, e a medida dos níveis séricos de BDNF e TrkB, por imunoensaio enzimático (Kit ELISA). O grau de compensação do metabolismo dos carboidratos foi avaliado pelo nível de glicemia de jejum e hemoglobina glicada (HbA1c), usando o método de troca iônica por microcoluna, certificado de acordo com o *National Glycohemoglobin Standardization Program* (NGSP) ou a *International Federation of Clinical Chemistry* (IFCC), e padronizado de acordo com os valores de referência adotados no *Diabetes Control and Complications Trial* (DCCT).

O ensaio de imunoabsorção enzimática em sanduí­che (Kit ELISA) foi realizado para cálculo quantitativo do fator neurotrófico cerebral e sua alta afinidade para receptor de tropomiosina quinase no soro, utilizando sistemas de teste da Cloud-Clone Corp (EUA). O conjunto de microplacas continha anticorpos conjugados com biotina, e específicos para BDNF e TrkB. As amostras foram adicionadas a poços específicos da placa. Em seguida, foi adicionado a cada poço da placa um conjugado de peroxidase-avidina de raiz forte, e incubado. Após a adição do substrato enzimático, a cor mudou apenas nos poços contendo BDNF ou TrkB, anticorpos com biotina e peroxidase-avidina de raiz forte. A reação enzimática foi interrompida pela adição de uma solução de ácido sulfúrico. A densidade óptica foi medida pelo método fotométrico, com um comprimento de onda de 450±10nm. A concentração de BDNF ou TrkB em amostras foi calculada de acordo com a escala de calibração padrão, preparada de acordo com os resultados da análise em uma série padrão. Conforme as instruções para ELISA, as concentrações da curva padrão foram 10ng/mL, 5ng/mL, 2,5ng/mL, 1,25ng/mL, 0,625ng/mL, 0,312ng/mL, e 0,156ng/mL.^(^^22^^)^ A dose mínima detectável de TrkB foi inferior a 0,058ng/mL, e a faixa de detecção foi de 0,156-10ng/mL. A dose mínima detectável de BDNF foi inferior a 0,011ng/mL, e a faixa de detecção foi de 0,031-10,0ng/mL. Os níveis séricos de BDNF e TrkB obtidos foram expressos como ng/mL.

### Análise estatística

A análise estatística foi realizada utilizando o programa Statistica versão 10. Os métodos estatísticos descritivos foram usados para calcular médias e desvios padrão, e métodos não paramétricos, em particular o teste em U de Mann-Whitney, para comparar duas amostras independentes de dois grupos independentes. O limiar p<0,05 foi usado para avaliar a probabilidade da hipótese nula. As relações entre vários fatores foram analisadas através do coeficiente de correlação Spearman. O nível crítico de significância no teste de hipóteses estatísticas foi considerado igual a 0,05. A influência de diversas variáveis independentes sobre a variável dependente foi verificada pela análise multivariada de variância (Anova). A qualidade do modelo foi determinada calculando R2, o quadrado do coeficiente de correlação múltipla; o limiar p<0,05 foi usado para avaliar a significância estatística da hipótese nula.

## RESULTADOS

O metabolismo dos carboidratos em pacientes com PND estava descompensado. A média de glicemia de jejum foi 9,01±4,09mmol/l, e excedeu significativamente o Grupo Controle (4,78±0,57mmol/l, U=5,615, p=0,000). A média de hemoglobina glicada foi 7,62±2,11%, que é estatisticamente maior do que no grupo de indivíduos saudáveis (5,04±0,36%, U=4,143, p=0,000).

As queixas mais frequentes apresentadas pelos pacientes do Grupo Pacientes foram dormência, parestesia, instabilidade ao caminhar, e cãibras noturnas. Sinais de dor estavam presentes em mais da metade dos pacientes (44; 62,8%), e 32 deles tinham um componente neuropático (45,7%). Outros participantes do estudo que relataram sintomas de dor tinham um caráter nociceptivo, representado principalmente por dor musculoesquelética, dor lombar e dor de cabeça. A pontuação média de intensidade de dor pela NRS em pacientes do Grupo Pacientes foi 4,58±3,21 pontos, que é significativamente maior do que no Grupo Controle (0,93±1,73 pontos, U=3,798, p=0,000). A intensidade média da dor neuropática, de acordo com a escala PainDETECT, no Grupo Pacientes foi de 13,40±10,38 pontos, que também foi significativamente maior do que o valor médio do Grupo Controle, ou seja, 2,50±0,85 pontos (U=6,348, p=0,000).

O exame neurológico mostrou sinais de PND pronunciada em 53 pacientes do Grupo Pacientes (75,7%), e sinais graves de polineuropatia foram diagnosticados em 17 deles (24,3%). No Grupo Controle, nenhuma anormalidade foi observada no sistema nervoso periférico, ao exame clínico. O valor médio d e NDS em pacientes com PND foi 14,98±7,12 pontos, maior que no Grupo Controle (0,50±0,76 pontos, p=0,000, U=5,948).

Na ENMG, todos os pacientes do Grupo Pacientes apresentaram sinais de polineuropatia axonal ou desmielinizante simétrica variável, de moderada (59 casos, 84,3%) a grave (11 casos, 15,7%).

Em pacientes com PND, o nível sérico médio de BDNF determinado por ELISA foi significativamente maior do que no Grupo Controle (3,44±2,67ng/mL *versus* 1,07±0,64ng/mL, p=0,000). O nível sérico de TrkB em pacientes com PND também foi significativamente maior do que no Grupo Controle (4,65±2,56ng/mL *versus* 1,66±0,63ng/mL, p=0,000).

Todos os pacientes com PND foram divididos em dois grupos com base na presença de dor neuropática, de acordo com o desenho do estudo. O primeiro grupo incluiu 32 pacientes com dor neuropática, com escore médio no PainDETECT de 24,51±3,16 pontos, e médio na NRS de 7,05±2,71 pontos. Todos os pacientes do primeiro grupo queixaram-se de sintomas típicos de dor neuropática, localizados simetricamente nas partes distais de ambos os pés. Em alguns pacientes, juntamente com dano aos nervos das pernas, os membros superiores também estavam envolvidos no processo patológico. Um total de 38 pacientes sem dor neuropática foi incluído no segundo grupo; o escore médio do PainDETECT foi 7,09±5,01, que foi significativamente menor do que no primeiro grupo. A pontuação média de MNSI no primeiro grupo excedeu significativamente o parâmetro correspondente para pacientes com uma forma indolor de neuropatia, como mostrado na tabela 1. Além disso, a avaliação dos sintomas subjetivos pela escala NSS em pacientes com dor neuropática foi significativamente mais pronunciada do que em pacientes com uma forma indolor (Tabela 1).

**Tabela 1:** Parâmetros clínicos e neurofisiológicos de pacientes com e sem dor

Critérios	Grupo 1 Média ±DP	Grupo 2 Média ±DP	Valor de U	Valor de p
Idade, anos	59,94±11,04	58,78±12,37	0,292	0,769
Índice de massa corpórea, kg/m^2^	33,11±6,08*	29,49±4,88*	2,791*	0,005*
Duração de *diabetes mellitus*, anos	11,00±8,64	10,40±9,48	0,418	0,675
Glicemia em jejum, mmol/l	8,71±2,69	8,92±4,73	0,993	0,32
HbA1c, %	7,45±2,21	7,35±2,43	0,296	0,767
NRS, pontos	7,05±2,71*	2,60±2,17*	3,911*	0,000*
PainDETECT, pontos	24,51±3,16*	7,09±5,01*	7,355*	0,000*
MNSI, pontos	6,85±2,06*	4,71±2,31*	3,618*	0,000*
NSS, pontos	6,70±2,55*	3,60±2,16*	3,045*	0,002*
NDS, pontos	15,88±7,71	14,75±7,49	2,445	0,064
S-amplitude nervo sural, mV	2,83±3,73	2,68±3,76	-0,087	0,930
Latency nervo sural, ms	2,82±1,79	2,87±2,49	0,185	0,852
NCVnervo sural, m/s	30,03±18,50	35,24±16,52	0,185	0,773
M-amplitude nervo fibular, mV	1,96±1,95	1,98±2,04	0,078	0,937
Latency nervo fibular, ms	3,55±0,96	4,02±1,44	-1,351	0,176
NCV, nervo fibular, m/s	38,45±7,9	29,85±11,25	-0,782	0,434
BDNF, ng/mL	4,14±2,75*	2,31±1,31*	2,171*	0,029*
TrkB, ng/mL	3,11±1,76*	4,72±2,81*	3,541*	0,000*

* Os valores significativos com p<0,05.

HbA1c: hemoglobina glicada; NRS: *Numerical Rating Scale*; MNSI: *Michigan Neuropathy Screening Instrument*; NSS: *Neurological Symptoms Score*; NCV: velocidade de condução nervosa; BDNF: fator neurotrófico derivado do cérebro; TrkB: receptor de tropomiosina quinase B; DP: desvio padrão.

Em ambos os grupos, os pacientes não diferiram em idade, duração do *diabetes mellitus*, glicemia de jejum, e hemoglobina glicosilada. Os pacientes com forma dolorosa de PND tinham sobrepeso (33,11±6,08kg/m^2^) em contraste com os pacientes com forma indolor (29,49±4,88kg/m^2^, p=0,005). A gravidade da polineuropatia pelo NDS também não diferiu em ambos os grupos. Não foram encontradas diferenças nos parâmetros da ENMG nos dois grupos (Tabela 1).

No primeiro grupo, os níveis séricos de BDNF (4,14±2,75ng/mL) foram significativamente maiores do que no segundo grupo (2,31±1,31ng/mL, p=0,001). Entretanto, os níveis séricos de TrkB no primeiro grupo (3,11±1,76ng/mL) foram significativamente menores do que no segundo grupo (4,72±2,81ng/mL, p=0,005).

A análise de correlação revelou interdependências de IMC e gravidade da dor por NRS (R=0,315, p=0,001) e por uma escala PainDETECT (R=0,327, p=0,001).

De acordo com os resultados da análise estatística, a idade e o sexo dos pacientes não interferiram na concentração do fator neurotrófico e receptor (p>0,05). O excesso de peso foi associado à alta expressão sérica de BDNF (R=0,412, p=0,001). Foi observada a correlação inversa do IMC e TrkB level (R=-0,345, p=0,001).

Foi encontrada uma correlação entre gravidade da dor neuropática por PainDETECT e aumento da BDNF circulante (R=0,392, p=0,012), e diminuição do TrkB sérico (R=-0,354, p=0,015), como mostrado na figura 1 e 2.

**Figura 1:** Correlação entre o PainDETECT e o fator neurotrófico derivado do cérebro. Nível de dor: a curva sérica do fator neurotrófico derivado do cérebro é mostrada no painel superior direito através de um gráfico de dispersão. A dor neuropática foi avaliada usando a escala PainDETECT. O eixo “y” mostra os níveis séricos do fator neurotrófico derivado do cérebro. O fator neurotrófico derivado do soro do cérebro foi medido por ELISA. Os níveis séricos do fator neurotrófico derivado do cérebro foram expressos em ng/mL

**Figura 2:** Correlação entre PainDETECT e o receptor de tropomiosina quinase B. PainDETECT: o gráfico de dispersão do receptor de tropomiosina sérica quinase B é mostrado no painel superior direito e o nível do receptor de tropomiosina quinase B é mostrado no eixo “y” e PainDETECT no eixo “x”. O receptor sérico de tropomiosina quinase B foi medido por ELISA. Os níveis séricos do receptor de tropomiosina quinase B foram expressos em ng/mL

A glicemia de jejum foi correlacionada com níveis séricos de BDNF (R=0,327, p=0,001). Foi encontrada uma correlação direta entre a hemoglobina glicada e os níveis séricos de TrkB (R=0,264, p= 0,001).

Observou-se uma relação direta entre os níveis de BDNF e amplitude S do nervo sural (R=0,263, p=0,016). Os níveis séricos de TrkB foram associados mais explicitamente com sinais de ENMG de comprometimento da condução do nervo. Foi demonstrada uma correlação direta entre o nível de TrkB e a latência do nervo fibular em membros distais (R=0,311, p=0,001), assim como a relação inversa na amplitude S do nervo sural (R=-0,235, p=0,023) e amplitude M do nervo fibular (R=-0,246, p=0,015, p=0,01). Além disso, os níveis séricos de TrkB correlacionaram com baixo NCV por ENMG (R=-0,574, p=0,001).

Analisamos os fatores associados à presença e gravidade da dor neuropática pelo modelo Anova. Os níveis séricos de TrkB foram divididos em baixos (<3,0ng/mL) e altos (>3,0ng/mL), e os níveis de BDNF em baixos (<2,0ng/mL) e altos (>2,0ng/mL). A obesidade foi diag­nosticada como IMC acima de 30kg/m^2^, de acordo com os critérios da OMS. Estimamos a intensidade da dor usando a escala PainDETECT. Como mostrado na figura 3, pacientes obesos com baixos níveis séricos de TrkB e hiperexpressão de BDNF sérico apresentaram maior intensidade de dor neuropática. O significado da hipótese foi avaliado, a força dos efeitos foi suficiente (R2=0,728) e o modelo foi estatisticamente signifi­cativo (p=0,004).

**Figura 3:** Modelo preditor de dor neuropática. A influência de diversas variáveis independentes sobre a variável dependente foi verificada pela Anova. A qualidade do modelo foi avaliada calculando-se R2, o quadrado do coeficiente de correlação múltipla, e o limiar p <0,05 foi usado para avaliar a significância estatística da hipótese nula. As barras verticais denotam intervalos de confiança de 95%. O painel esquerdo mostra gráficos da dependência do nível de dor na escala PainDETECT nos níveis séricos do receptor de tropomiosina quinase B e do fator neurotrófico derivado do cérebro, na obesidade. A escala PainDETECT é classificada de 0 a 28 e exibida no eixo “y”. Os níveis séricos do receptor de tropomiosina quinase B são divididos em níveis altos (>3,0ng/mL) e baixos (<3,0ng/mL) e ocupam o eixo “x”. A curva azul mostra altos níveis séricos de fator neurotrófico derivado do cérebro (>2,0ng/mL). A curva vermelha corresponde a baixos níveis séricos de fator neurotrófico derivado do cérebro (<2,0ng/mL). O painel direito mostra a relação dos níveis séricos do receptor da tropomiosina quinase B, representada no eixo “x”, intensidade da dor por PainDETECT no eixo “y”, e níveis de neurotrofina derivada do cérebro (curva vermelha - fator neurotrófico derivado do cérebro <2,0ng/mL, curva azul - fator neurotrófico derivado do cérebro >2,0ng/mL) em casos de peso corporal normal. O significado estatístico dos modelos resultantes foi alcançado, e o efeito atual foi suficiente R2=0,728, p=0,004.

## DISCUSSÃO

A dor neuropática foi o principal sinal clínico de PND em 45,7% dos casos, de acordo com os resultados do estudo. Os níveis séricos de BDNF apresentaram forte correlação com a presença e intensidade da dor neuropática. O fator neurotrófico derivado do cérebro atua como um ativador da sensibilização central da dor, tem um efeito direto na modulação da dor neuropática, e modula sua gravidade.

Este estudo avaliou, pela primeira vez, os níveis séricos de TrkB em pacientes com polineuropatia diabética. Ao contrário de seu bioligante, os níveis de TrkB estão inversamente relacionados à intensidade da dor neuropática. Além disso, os níveis séricos de TrkB apresentaram forte correlação com a gravidade da desmielinização das fibras nervosas na ENMG.

Comparamos essas correlações e os dados sobre a localização transmembrana do receptor na mielina dos nervos periféricos, bem como uma presença impossível da forma ativa livre do receptor no sangue.^(^^23^^,^^24^^)^ Tais fatos permitiram concluir que a detecção de TrkB no soro pode indicar a circulação de um receptor não funcionante.

Como discutido anteriormente, um dos mecanismos do efeito sensibilizador central do BDNF é a ativação da germinação sináptica de finas fibras C sensoriais no corno posterior, que leva à hiperexcitação dos receptores glutamatérgicos N-metil-D-aspartate (NMDA), ao desenvolvimento do fenômeno de desaferenciação, e a não inibição dos neurônios das vias somatossensoriais.^(^^25^^–^^28^^)^ De acordo com os resultados do estudo, este efeito se deve à interação do fator neurotrófico cerebral, precisamente com seu receptor de alta afinidade funcionalmente ativo, TrkB. A ativação do receptor quinase desencadeia sinais intracelulares que fornecem efeitos de neuroplasticidade e germinação de fibras sensoriais.^(^^29^^,^^30^^)^ Além disso, a germinação de fibras com várias funcionalidades sensíveis leva ao desenvolvimento de parestesia, alodinia e hiperpatia.^(^^28^^,^^30^^)^ Assim, a sinalização BDNF-TrkB está envolvida no desenvolvimento da dor neuropática na PND, e é responsável pelos efeitos da hiperalgesia e transformação da dor nociceptiva em dor neuropática.

São necessárias mais pesquisas para estudar as possibilidades de controlar a cascata de sinais intracelulares, ativados pelo receptor de tropomiosina quinase B, no desenvolvimento da síndrome da dor neuropática. As descobertas científicas nesta área permitirão definir novos vetores no alívio da dor em polineuropatia.

## CONCLUSÃO

O estudo demonstrou que a forma dolorosa de neuropatia é mais típica para pacientes obesos. Demonstrou que o excesso de peso está negativamente correlacionado com níveis mais altos de receptores séricos de tropomiosina quinase B. Assim, supomos que o fator neurotrófico derivado do cérebro e seus receptores de alta afinidade sejam responsáveis pela modulação da obesidade. Como discutido anteriormente, os processos inflamatórios e as vias da obesidade desempenham um papel importante na patogênese da polineuropatia diabética. A ativação do sistema BDNF-TrkB pode ser um elo no desenvolvimento de hiperalgesia, obesidade, e inflamação na forma dolorosa de polineuropatia diabética, e esta ligação é muito provavelmente feita pela via de sinalização do fosfatidilinositol-3 quinase/Akt (PI3K/Akt). É necessário um estudo mais detalhado das vias específicas de ativação intracelular no desenvolvimento e integração do excesso de peso, dor neuropática e sistema imunológico.

As medidas periféricas do fator neurotrófico derivado do cérebro e do receptor da tropomiosina quinase B fornecem as bases para estudar a patogênese da dor neuropática na polineuropatia diabética, e sua ação como biomarcadores da doença. Vários níveis séricos podem ajudar a diferenciar a dor e formas indolores de polineuropatia diabética.
